# Members of the murine *Pate *family are predominantly expressed in the epididymis in a segment-specific fashion and regulated by androgens and other testicular factors

**DOI:** 10.1186/1477-7827-9-128

**Published:** 2011-09-26

**Authors:** Heikki T Turunen, Petra Sipilä, Dwi Ari Pujianto, Anastasios E Damdimopoulos, Ida Björkgren, Ilpo Huhtaniemi, Matti Poutanen

**Affiliations:** 1 Department of Physiology, Institute of Biomedicine, University of Turku, Kiinamyllynkatu 10, FIN-20520, Turku, Finland; 2 Turku Graduate School of Biomedical Sciences, Kiinamyllynkatu 13, FIN-20520, Turku, Finland; 3 Turku Center for Disease Modeling, Kiinamyllynkatu 10, FIN-20520, Turku, Finland; 4 Institute of Reproductive and Developmental Biology, Imperial College London, Hammersmith Campus, London W12 0NN, UK; 5 Department of Biology, Faculty of Medicine, University of Indonesia, Jakarta Pusat, Indonesia

## Abstract

**Background:**

Spermatozoa leaving the testis are not able to fertilize the egg *in vivo*. They must undergo further maturation in the epididymis. Proteins secreted to the epididymal lumen by the epithelial cells interact with the spermatozoa and enable these maturational changes, and are responsible for proper storage conditions before ejaculation. The present study was carried out in order to characterize the expression of a novel *Pate *(prostate and testis expression) gene family, coding for secreted cysteine-rich proteins, in the epididymis.

**Methods:**

Murine genome databases were searched and sequence comparisons were performed to identify members of the *Pate *gene family, and their expression profiles in several mouse tissues were characterized by RT-PCR. Alternate transcripts were identified by RT-PCR, sequencing and Northern hybridization. Also, to study the regulation of expression of *Pate *family genes by the testis, quantitative (q) RT-PCR analyses were performed to compare gene expression levels in the epididymides of intact mice, gonadectomized mice, and gonadectomized mice under testosterone replacement treatment.

**Results:**

A revised family tree of *Pate *genes is presented, including a previously uncharacterized *Pate *gene named *Pate-X*, and the data revealed that *Acrv1 *and *Sslp1 *should also be considered as members of the *Pate *family. Alternate splicing was observed for *Pate-X, Pate-C *and *Pate-M*. All the *Pate *genes studied are predominantly expressed in the epididymis, whereas expression in the testis and prostate is notably lower. Loss of androgens and/or testicular luminal factors was observed to affect the epididymal expression of several *Pate *genes.

**Conclusions:**

We have characterized a gene cluster consisting of at least 14 expressed *Pate *gene members, including *Acrv1, Sslp1 *and a previously uncharacterized gene which we named *Pate-X*. The genes code for putatively secreted, cysteine-rich proteins with a TFP/Ly-6/uPAR domain. Members of the *Pate *gene cluster characterized are predominantly expressed in the murine epididymis, not in the testis or prostate, and are regulated by testicular factors. Similar proteins are present in venoms of several reptiles, and they are thought to mediate their effects by regulating certain ion channels, and are thus expected to have a clinical relevance in sperm maturation and epididymal infections.

## Background

Spermatozoa leaving the testis are immotile while their epididymal maturation is known to be essential for the attainment of progressive motility, and for the abilities to recognize and fertilize the oocyte [1,2]. This maturation process is associated with interaction between spermatozoa and epididymal luminal fluid that bring about changes in sperm membrane structure through removal, addition, and reorganization of the lipid bilayer of the plasma membrane of spermatozoa [3]. Studies have shown that each epididymal region has a distinct function governed by region-specific gene expression [4-7]. Protein families with highly segment-specific expression in the epididymis include, for example, defensins, lipocalins, proteases, protease inhibitors, proteins with a disintegrin and metallopeptidase domain (ADAMs) and cysteine-rich secretory proteins (CRISPs) [8-12]. Region-specific expression of members of these families is thought to be, at least partially, responsible for creating the region-specific luminal environment essential for epididymal sperm maturation.

Interestingly, several cysteine-rich secreted epididymal proteins, especially CRISPs, have also been identified in venoms of various reptiles [13]. The disulfide bridges between the conserved cysteine residues are thought to help maintaining the structure of the venom proteins in the hostile environment of the target prey's bloodstream [14,15]. Similarly, the dehydrated epididymal luminal fluid is a challenging environment for proteins, and, thus, stability provided by disulfide bridges may be essential for maintaining protein function [16]. Based on their predicted structural properties these cysteine-rich proteins may participate in non-specific defense mechanisms against micro-organisms in the epididymal lumen and/or maturation of the spermatozoa via regulating the activities of certain ion channels, although the exact molecular mechanisms responsible for their functions are still poorly known.

Recently two research groups have independently identified a genomic locus from various mammalian species, including human, with several genes encoding predicted secreted proteins with a cysteine-rich TFP/Ly-6/uPAR domain [15,17]. The gene family was named *Anlp *(α-neurotoxin-like protein) and *Pate *by the two groups. The TFP/Ly-6/uPAR domain has 8-10 highly conserved cysteine residues, but their distribution differs from that of cysteine-rich domain of CRISPs [18]. The cysteines of known PATE proteins form two motifs: C[XX]C[X^7-8^]C[X^6^]C[X^7-8^]C and C[X^3^]C[X^15-16^]CC[X^4-5^]CN, where X stands for any amino acid and the superscripts denote their number, and the N stands for asparagine, which is also conserved. Similarly to CRISPs, the TFP/Ly-6/uPAR domain is identified in snake toxins [19], but the domain is present in certain membrane receptors as well [20,21]. Furthermore, the domain was found in murine Ly-6 proteins, and is, thus, termed the TFP/Ly-6/uPAR domain [18]. It confers no known enzymatic activity but binds to a wide range of cell surface receptors, ion channels and enzymes [22,23]. Of PATE proteins mouse (m) PATE-B has been shown to inhibit Ca^2+ ^uptake of spermatozoa [24], and mPATE-C, mPATE-P and human (h) PATE-B modulate nicotinic acetylcholine receptors (nAChRs) [17].

Most of the *Anlp*/*Pate *family members have been reported to be predominantly expressed in the prostate and the testis, whereas the UniGene entries show high epididymal expression. In the present study we studied the expression of several *Pate *family genes in the male reproductive organs with a particular focus in the epididymis, and provide evidence of their regulation by androgens and other testicular factors. In addition, by comparing published data of the genes to the current annotations in the Ensembl and NCBI databases, a putative novel member of the family was discovered.

## Methods

### Identification of the *Pate*-family members and analyses *in silico*

Bioinformatic tools at Ensembl [25] and the National Center for Biotechnology Information (NCBI) [26] websites were used to study annotated and predicted genes in mouse chromosome 9 at location A4. The annotations and predicted PATE-family protein sequences were compared with the mouse genomic databases and to sequences published previously [15,17] by BLAST [27]. The ClustalW program [28] was used to align the identified amino acid sequences, the signal peptide cleavage sites were identified with SignalP 3.0 [29], and polyadenylation sites were identified with the Poly(A) Signal Miner program [30]. Phylogenetic trees including PATE-family members were constructed with the MEGA4 program [31].

### Experimental animals and RNA extraction

C57BL/6N male mice were used throughout the study (Harlan Laboratories, Inc., Indianapolis, IN). All animal handling was conducted in accordance with Finnish Animal Ethics Committee and the Institutional animal care policies of the University of Turku (Turku, Finland), which fully meet the requirements as defined in the NIH Guide on animal experimentation. The mice were housed under controlled environmental conditions (12 h light/12 h darkness, temperature 21 ± 1°C) and fed with standard pelleted chow and tap water *ad libitum*. To analyze the tissue distribution of gene expression, various tissues were isolated from 7-8 week-old mice for RNA extraction. To analyze the androgen dependency of gene expression, 12 sexually mature male mice (divided into 4 groups of 3 mice) were anesthetized by an intraperitoneal injection of 400-600 μl 2.5% Avertin (2-2-2 tribromoethanol, Aldrich Chemical Co., Milwaukee, WI). Three groups (nine mice) were gonadectomized, while the fourth group served as controls. Testosterone (T) treatment was given to one group of gonadectomized mice by subcutaneous 1 cm long SILASTIC silicon tubes (Dow Corning, Inc., Midland, MI; inner diameter = 1.98 mm, outer diameter = 3.18 mm) filled with T powder (Sigma-Aldrich Corp., St. Louis, MO). The treatment provides T levels above the normal level of WT male mice [32]. The proximal epididymides were collected 8 hours and 1 day after gonadectomy from the non-treated mice, and after 7 days of T-treatment, and from the un-operated control group. Total RNA was isolated using the TRI Reagent (Life Technologies Corporation, Carlsbad, CA).

### RT-PCR and qRT-PCR

RT-PCR was performed to analyze the tissue distribution of *Pate *family gene expression. The tissues studied are listed in Table 1. One μg of total RNA from each tissue was DNase treated with Amplification Grade DNase I (Invitrogen, Carlsbad, CA) and reverse transcribed with DyNAmo cDNA Synthesis Kit (FinnZymes, Espoo, Finland). Biotools DNA polymerase (Biotools, Madrid, Spain) was used for PCR. For *Pate-C *primer pair Pate-C F1-Pate-C R1 and for *Pate-M *primer pair Pate-M F1-Pate-M R1 were used. All primer sequences and specific reaction conditions are presented in Table 2. Sequencing of the PCR products was performed at the Finnish Microarray and Sequencing Centre (Turku, Finland). For *Pate-M *qRT-PCR primer pair Pate-M F3-Pate-M R3 was used. To analyze regulation of gene expression by androgens qRT-PCR analyses were performed. All qRT-PCR analyses were done in triplicates. DNase treatment and cDNA synthesis was carried out as described above, and DyNAmo Flash SYBR Green (FinnZymes) was used for qPCR. The relative standard curve method was used to calculate the gene expression levels, and qRT-PCR results on *L19 *were used for normalization. All statistical analyses were performed by using the SigmaPlot program (Systat Software Inc., Point Richmond, CA). Gene expression levels were compared with Student's t test, and standard deviations are shown as error bars.

**Table 1:** Expression of *Pate *family genes in the mouse

	1	2	3	4	5	6	7	8	9	10	11	12	13	14	15	16	17	18	19	20	21	22	23	24	25
***Pate***	-	-	-	-	-	-	-	-	-	-	-	-	-	-	-	-	-	-	-	-	++	++	-	-	-
***Pate-A***	-	-	-	-	-	-	-	-	-	-	-	-	-	-	-	-	-	-	-	-	-	+++	+	-	-
***Pate-B***	+	-	+	-	-	-	-	-	++	+	-	+	-	-	+	-	-	-	-	+	+++	+++	+++	+++	++
***Pate-C***	-	-	-	-	-	-	-	-	-	-	-	-	-	-	-	-	-	-	-	-	+++	+++	++	-	-
***Pate-DJ***	-	-	-	-	-	-	-	-	-	-	-	-	-	-	-	-	-	-	-	-	-	+++	++	-	-
***Pate-E***	+	-	-	+	-	-	-	-	-	-	-	-	-	-	-	-	+	-	-	+	++	+++	++	-	-
***Pate-H***	+	-	-	-	-	-	-	-	-	-	-	+	-	-	+	++	-	+	++	-	+++	+++	-	+++	++
***Pate-M***	+	+	+	-	-	++	++	++	+	++	++	++	-	-	-	-	+	++	++	+++	+++	++++	++++	-	-
***Pate-N***	-	-	-	-	-	-	-	-	-	-	-	-	-	-	-	-	-	-	+	-	-	+++	-	-	-
***Pate-P***	-	-	-	-	-	-	-	-	-	-	-	-	-	-	-	-	-	-	-	-	-	-	-	-	-
***Pate-Q***	-	-	-	-	-	-	-	-	-	-	-	-	-	-	-	-	-	-	-	++	-	++	-	-	-
***Pate-X***	-	-	-	-	-	-	-	-	-	-	-	-	-	-	-	-	-	-	-	-	-	+++	-	+	+
***Sslp1***	-	-	-	-	-	-	-	-	-	-	-	-	-	-	-	-	-	-	-	-	++	++	+++	+++	+++
***Acrv1***	-	-	-	-	-	-	-	-	-	-	-	-	-	-	++	-	-	-	-	++++	+++	+++	+	+++	+

Expression profiles of *Pate *family members based on RT-PCR results. Levels of expression were subjectively graded based on the intensity of the PCR band. Detectable expression is graded from + to ++++, where + indicates barely detectable and ++++ very strong expression. Dash (-) indicates no detectable expression. 1, pituitary; 2, adrenal gland; 3, thymus; 4, liver; 5, lymph node; 6, brain; 7, salivary gland; 8, lung; 9, stomach; 10, small intestine; 11, kidney; 12, large intestine; 13, heart; 14, pancreas; 15, eye; 16, skeletal muscle; 17, spleen; 18, bladder; 19, bulbourethral gland; 20, testis; 21, seminal vesicle; 22, epididymis; 23, vas deferens; 24, dorsal prostate; 25, ventral prostate.

**Table 2:** Primers used in the study

Primer	**T**_**M **_**(°C)**	Sequence
Pate-A F1	55	ACTGACCGTCCTGAGCACTT
Pate-A R1	55	TAGCTTGGACTGTGTGTGAGA
Pate-B F1	59	AATCAGCACACTGCTCATCG
Pate-B R1	59	CGAGCACATTTGCTTTGAGT
Pate-C F1	55	TCCTGAGGCTGTGTCTCTTTC
Pate-C R1	55	TTCGTGCACTTAGTCTCAGCA
Pate-C R2	55	TCCAGATCTTTCTTCTGTGACG
Pate-C F3	59	TGTATGATCCGCAGAACCTG
Pate-C R3	59	AAGGAAAGGGCTGATGAGGT
Pate-DJ F1	58	TGTGTAACATGCCACCTTCG
Pate-DJ R1	58	TGATAACTGGAGAGAGCCACTG
Pate-E F1	62	AGCTGAGCATCGTTCTGCTA
Pate-E R1	62	GGCATCTAGAGTATGCATCATTTG
Pate-H F1	59	CCGGTGACAAAAATCAGTACA
Pate-H R1	59	TGGAACCCATACATGAACCTG
Pate-M F1	59	GACAAGGGTTGCAGGATGTT
Pate-M R1	59	CAGCGCAGGTCTGTCTATGA
Pate-M F2	59	GAGCCATTCATGCAAAACCT
Pate-M R2	59	CGATGATCAATCCGTGAAGT
Pate-M F3	57	ACCTGGAGGCAGGACTCATA
Pate-M R3	57	GTGGACGTGTCTGTGGAGAA
Pate-N F1	55	GTCTCATTCAATGGGGGAAC
Pate-N R1	55	TAGCTTTCATTGCAGCAGGA
Pate-P F1	59	CTTTGCTGGTGATGTCCCTG
Pate-P R1	59	CTGTCTTATCTCCAATCATA
Pate-Q F1	59	TCCTGTCTTTGCTGGTGATG
Pate-Q R1	59	GAGCACCCAACAACATATGAAA
Pate-X F1	55	GGATGTAGGAGAAAGAGTGCTGA
Pate-X R1	55	CAGGTGCACAGGGTTTACAA
Pate-X F2	57	GTCAGAAGGAGGCCCAATTA
Pate F1	59	GATGCCTCTATCTTTCTGTGC
Pate R1	59	TCCTCTTCCTCTGGTGCAAT
Acrv1 F1	55	GACGAAGCAGGTGAACAGGT
Acrv1 R1	55	ACCCTTGAACCATGAACTGG
Sslp1 F1	55	ACTCTTGGGCATCTTTTTGC
Sslp1 R1	55	AGACATCCCTGGAAGCCATA

### Northern hybridization

For Northern hybridization 20 μg of total RNA or 4 μg of poly-A mRNA extracted from different segments of the epididymis were denatured, separated on a 1% denaturing agarose gel, and transferred onto nylon membrane (Hybond-XL, Amersham Biosciences, Buckinghamshire, UK). Probes for detecting the *Pate-C *mRNAs were generated by RT-PCR using primer pair Pate-C F3-Pate-C R3 (Table 2) from total RNA of caput epididymidis and labeled with [^32^P]αCTP. Probe hybridization was detected by autoradiography using X-ray film (Fuji Photo Film Co. Ltd., Tokyo, Japan) or a phosphor imager (Fuji Photo Film Co. Ltd.).

## Results and discussion

### *Pate *gene family

**Figure 1:**
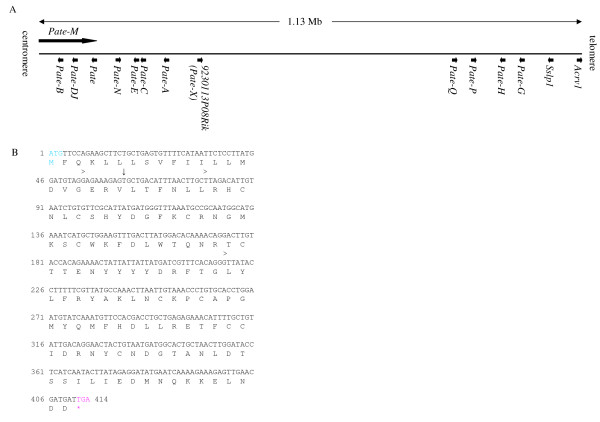
**The *Pate *gene family A**: A schematic representation of the mouse genomic locus of *Pate *genes. Arrows indicate direction of transcription. **B**: Nucleotide and predicted amino acid sequences of *9230113P08Rik *(*Pate-X*). Exon boundaries are marked by > and the predicted signal peptide cleavage site by ↓.

All known mouse *Pate *genes reside in a single cluster of 1.13 Mb in length, in chromosome 9 at location A4 (Figure 1A). Most members consist of three exons, although this is not a strictly conserved feature. The genes code for 98-125 amino acids (aa) long proteins (Table 3) containing a signal peptide sequence and the TFP/Ly-6/uPAR domain with a highly conserved distribution of 10 cysteine residues. In two studies, 14 [17] and 8 [15] expressed members of the family have been observed, including *Acrv1 *(acrosomal vesicle protein 1) [33] and *Sslp1 *(secreted seminal vesicle Ly-6 protein 1) [34], which also code for proteins containing the aforementioned features characteristic for PATE proteins and should, thus, also be considered as members of the family based on sequence similarities and phylogenetic analyses. However, as the names *Acrv1 *and *Sslp1 *are already established in literature, we do not propose their re-naming.

**Table 3:** Known mouse *Pate *genes

Pate-name	official symbol	alternate names	GenBank ID	protein size (aa)	human homologue
*Pate-A*	*9230110F15*	*Anlp3*	77080	117	
*Pate-B*	*Pate4*	*Svs7*	56872	99	*PATE4*
*Pate-C*	*D730048I06Rik*	*Anlp2*	68171	106	
*Pate-DJ*	*Pate3*		100312956	98	*PATE3*
*Pate-E*	*AV379335*		100312986	109	
*Pate-G*	*Pate-G*		100312948	109	
*Pate-H*	*Gm5615*	*Anlp5*	434396	101	
*Pate-M*	*Pate2*	*Anlp1*	330921	111	*PATE2*
*Pate-N*	*EU703628*		100312949	116	
*Pate-P*	*Gm9513*		671003	99	
*Pate-Q*	*Gm7257*		639025	99	
*Pate-X*	*9230113P08Rik*		77908	136	
*Pate*	*Pate*		100312987	125	*PATE1*
	*Sslp1*		235973	99	
	*Acrv1*	*Msa63, SP-10*	11451	261	*ACRV1*

**Figure 2:**
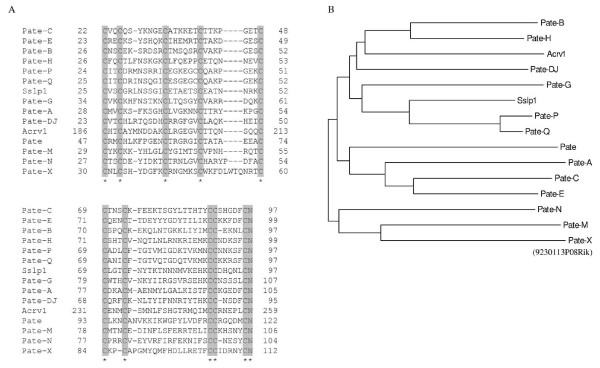
**Amino acid sequence comparisons of PATE proteins A**: The two cysteine containing motifs of PATE proteins. The asterisks mark conserved amino acids, and the numbers indicate the position of the amino acids counting from the N-terminal end. **B**: A phylogenetic tree based on full-length sequences of PATE family proteins.

In addition, our examination of the *Pate *genomic cluster revealed a previously uncharacterized gene *9230113P08Rik*. The current annotation in Ensembl indicates that the gene codes for a 93 aa long truncated PATE-like protein, lacking the signal peptide sequence and the three N-terminal cysteines characteristic for PATE proteins. However, we identified by RT-PCR and sequencing a transcript coding for a protein containing all defining PATE features (Figure 1B). The predicted full PATE-like protein is 136 aa in length and has a signal peptide sequence, and contains the ten conserved cysteines in a pattern closely resembling that of the other PATE proteins (Figure 2A). Phylogenetic analyses indicated a close evolutionary relationship between proteins coded by *9230113P08Rik *and other *Pate *genes (Figure 2B). Similarly, Ensembl annotations of orthologuous genes from several species, such as the rat, dog and macaque, indicated that the gene codes for a protein belonging to the PATE family. To maintain consistency in nomenclature, we named the gene as *Pate-X*.

Sequence comparisons and phylogenetic analyses of published *Pate *members and the current mouse genome sequences available in Ensembl and NCBI databases indicate that the gene known as *Pate-F *[17] (*Anlp4 *[15]) does not belong to the family. The gene codes for a protein containing only 8 cysteines, and lacks the conserved distribution of the cysteines characteristic for PATE proteins. Furthermore, *Pate-G *[17] expression was not observed in any tissues analyzed in the current study, although several different primer pairs and various PCR conditions were used. Thus, based on previous publications, current annotations and our experimental data, we have compiled a revised table of the murine *Pate *family, comprised of 15 expressed members (Table 3). The human orthologues identified in database search are indicated in Table 3.

Interestingly, both the genomic organization and structure of *Pate *genes resemble those of genes coding for toxin proteins in snakes. In mammals, many of these toxin-like proteins are thought to be involved in defence against micro-organisms ([15] and references therein). Snake toxin genes are under constant selective evolutionary pressure in order to help maintain the effectiveness of the venom. Likewise, mammalian antimicrobial peptide coding genes need to quickly adapt to the ever changing microbial flora. This may especially concern the environment of the epididymal lumen, which is not accessible to the immune cells due to physical barriers separating blood from the luminal contents, leaving the defence depending on anti-microbial peptides. Thus, to provide an extensive protection for the developing spermatozoa against micro-organisms, a large amount of rapidly evolving antimicrobial protein coding genes is necessary. Members of the *Pate *family may represent such genes.

### Alternate transcripts for *Pate*-family members

**Figure 3:**
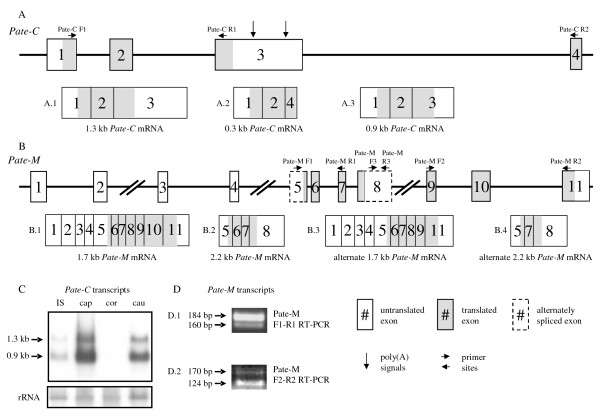
**Alternate *Pate-C *and *Pate-M *transcripts A**: A schematic figure of the *Pate-C *genomic locus and the detected *Pate-C *transcripts (A.1-A.3). Exon and intron lengths are not drawn to scale. **B**: A schematic figure of the *Pate-M *genomic locus and of the detected *Pate-M *transcripts (B.1-B.4). Exon and intron lengths are not drawn to scale. **C**: Northern hybridization of epididymal RNA with *Pate-C *specific probe revealing the presence of two transcripts. **D**: RT-PCR analysis of epididymal RNA with *Pate-M *specific primers as indicated in B. The longer PCR products reveal transcripts including exons 6 (top) and 10 (bottom), whereas the shorter products indicate their absence. The transcript sequences were further confirmed by sequencing the PCR products (not shown).

In addition to the transcripts coding for proteins with the defining PATE characteristics, the Ensembl database lists alternate protein coding transcripts for *Pate-C *and *Pate-M*, but not for other members of the family. However, for *Pate-X *Ensembl only shows a transcript coding for a truncated PATE-like protein, whereas we also detected by RT-PCR and sequencing another transcript containing an open reading frame for a complete PATE protein (Figure 1B). For *Pate-C *0.3 kb and 1.3 kb transcript sequences were predicted in Ensembl, and their presence was confirmed by experimental data. The two first exons of the transcripts are the same, whereas the third exons differ (Figure 3A). Only the 1.3 kb transcript codes for a protein with the ten cysteine residues characteristic for PATE proteins (Figure 3A.1), and furthermore, the 0.3 kb transcript (Figure 3A.2) was only barely detectable by qRT-PCR (data not shown). In addition, we discovered a novel 0.9 kb transcript by Northern hybridization (Figure 3A.3). The 0.9 kb transcript provided a stronger signal from epididymal total RNA than the 1.3 kb transcript (Figure 3C). However, no evidence for alternate splicing resulting to a 0.9 kb transcript was detected, and the Poly(A) Signal Miner program [30] identified three polyadenylation sites in the 1.3 kb mRNA, at positions 695, 699 and 1054 nucleotides from 5'-end, suggesting that the 0.9 kb transcript is produced by alternative usage of the polyadenylation sites rather than alternate splicing. Also, expressed sequence tag (EST) sequences corresponding to the transcripts produced by earlier polyadenylation are presented in the UniGene EST library. However, as full sequencing of the 0.9 and 1.3 kb transcripts was not performed, an element of uncertainty regarding the sequence of the 0.9 kb transcript remains.

For *Pate-M *the Ensembl database predicted the presence of two transcripts, 1.7 kb and 2.2 kb in length (Figure 3B), and the transcripts are comprised of 11 and 4 exons, respectively (Figure 3B.1 and 3B.2). The 2.2 kb transcript codes for a protein with all 10 cysteine residues characteristic for PATE proteins, while the protein coded by the 1.7 kb transcript lacks the C-terminal cysteine-doublet and the conserved CN pair. In addition, we detected two alternatively spliced isoforms by RT-PCR and sequencing, 2.2 kb and 1.7 kb in size. The alternative 2.2 kb transcript lacks exon six that results in a loss of eight amino acids, although no cysteines are lost and the reading frame is unaltered (Figure 3B.4). Furthermore, this transcript is similar to the other *Pate *family transcripts by being comprised of three exons, and codes for a protein retaining all PATE characteristics. An alternatively spliced isoform of the 1.7 kb transcript lacks the exon 10 that causes a deletion of 15 amino acids, and alters the reading frame at the C-terminal part of the protein (Figure 3B.3). However, the PATE signature domains are not affected. Of all the transcripts detected the 2.2 kb one containing four exons and all the conserved PATE elements is the predominant form. Although alternate protein coding transcripts for *Pate-C *and *Pate-M *are expressed, their expression levels in the male reproductive tract are clearly lower than those coding for proteins with all ten cysteine residues. Furthermore, even minor differences in the conserved distribution of cysteine residues will cause loss of sulfur bridges, and thus, alter the three-dimensional structure, potentially leading to major changes in the function. The highly conserved structures of *Pate *genes and proteins also likely lead to functional redundancy within the family, giving the opportunity of novel forms to evolve through mutations without compromising the existing functions of the proteins. The presence of these novel transcripts as well as their distinct expression patterns compared with the predominant transcripts may imply the occurrence of such evolution.

### *Pate*-genes are predominantly expressed in reproductive organs in male mice

**Figure 4:**
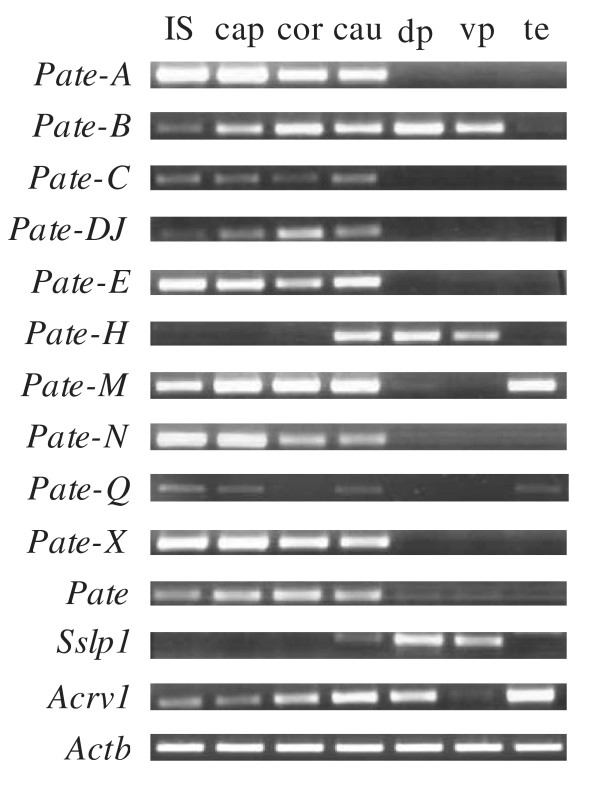
**Expression of *Pate *family genes in mouse epididymis, testis and prostate **Expression by RT-PCR after 36 amplification cycles. IS, initial segment; cap, caput; cor, corpus; cau, cauda; te, testis; dp, dorsal prostate; vp, ventral prostate. Samples with barely detectable expression as indicated in the text may not show visible bands in the figure.

We studied the expression of several *Pate *family members in 24 male mouse tissues, in different segments of the epididymis [initial segment (IS), caput, corpus and cauda] and dorsal and ventral prostate by RT-PCR. With the exceptions of placental *Pate-P *and *Pate-Q *the members studied were predominantly expressed in male reproductive organs, with *Pate, Pate-A, Pate-C, Pate-DJ, Pate-N, Pate-X *and *Sslp1 *showing no expression in non-reproductive organs. Expression in the testis was detected for *Pate-B *(barely detectable), *Pate-E *(barely detectable), *Pate-M, Pate-Q *and *Acrv1*, and in the prostate for *Pate-B, Pate-H, Pate-X *(barely detectable), *Sslp1 *and *Acrv1*. However, with the exception of *Pate-P *all the members studied were expressed in the epididymis, and the mRNA expression levels were also highest in the epididymis, except for *Pate-Q, Pate-B, Pate-H, Acrv1 *and *Sslp1*. Furthermore, several *Pate *members presented segment specific differences in the expression within the epididymis (Figure 4). The complete expression profiles for all analyzed *Pate*-genes are presented in Table 1.

The *Pate *family has been named based on their expression in the testis and prostate. However, the UniGene database suggested that the genes are expressed in the epididymis in mice rather than the testis or prostate, while the epididymis had not been included in all previous expression studies [17,33-35]. In the current study we have confirmed the predominant epididymal expression of *Pate *family genes. Furthermore, the expression in the testis or prostate was limited only to a minority of the members.

There is only limited experimental evidence for the functions of PATE proteins, but their strong expression in the epididymis and their resemblance to snake toxin proteins provide valuable information in this regard as well. Most toxin proteins exert their function by regulating the activities of certain ion-channels, and hPATE-B, mPATE-C and mPATE-P have been shown to affect through nACHRs [17], and mPATE-B to regulate Ca^2+ ^transport [24]. Although the sperm acrosome reaction has been shown to be mediated through nAChRs [36], the majority of acetylcholine effects in spermatozoa is thought to be carried out by muscarinic AChRs [37], indicating that the PATE proteins may not interact with the spermatozoa directly. However, hPATE-B has been shown to bind to spermatozoa, and due to mediation of Ca^2+ ^transport, a role in regulation of the acrosome reaction has been suggested [24]. Based on gene and protein similarities with toxins and anti-microbial properties of mammalian toxin-like proteins ([15] and references therein), it is also possible that the PATE proteins participate in defense against pathogens in the immune cell-free environment of the epididymal lumen. However, the anti-microbial properties of PATEs remain to be evaluated.

### Regulation of *Pate*-family gene expression by androgens and luminal factors

**Figure 5:**
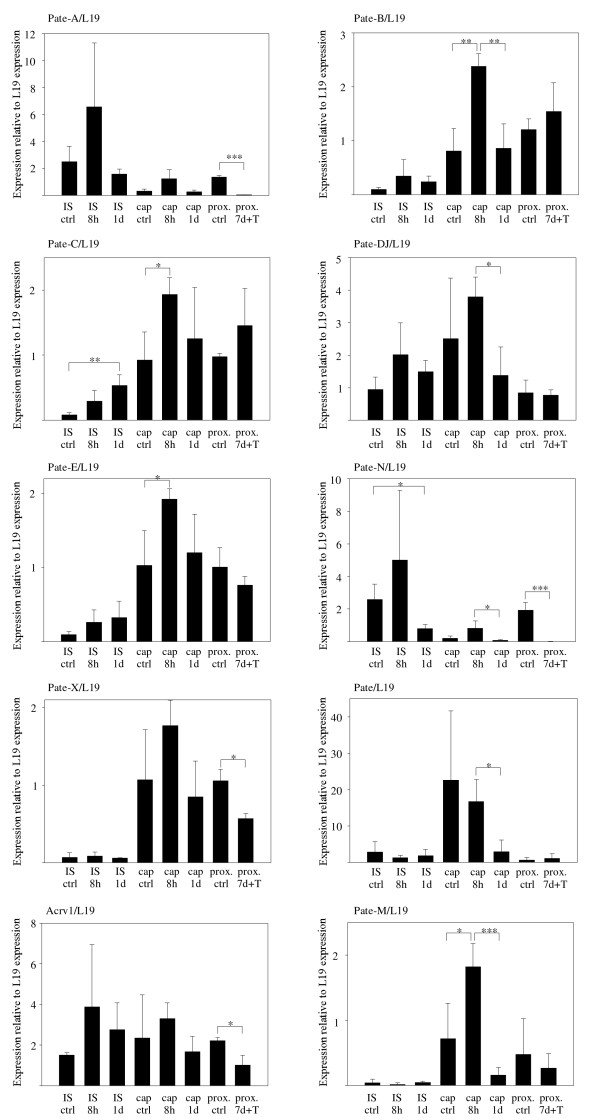
**Expression of *Pate *genes by qRT-PCR in intact, gonadectomized, and gonadectomized mice receiving testosterone treatment **Statistical significance of changes is indicated as follows: *, P < 0.05; **, P < 0.01; ***, P < 0.001. 8h, 8 hours after gonadectomy; 1d, 1 day after gonadectomy; 7d + T, 7 days after gonadectomy with testosterone treatment. Prox, proximal epididymis including the initial segment and caput; ctrl, control sample from intact mice.

Predominant expression of *Pate *family genes in the male reproductive organs suggests regulation by testicular factors, and *Pate-B, Pate-E *and *Sslp1 *have previously been reported to be regulated by androgens [17,34]. We further studied the effect of androgens and other testicular factors on *Pate *family members' expression by comparing expression levels in the IS and caput epididymidis of intact mice, gonadectomized mice and gonadectomized mice receiving testosterone treatment. Typically, genes expressed in the caput epididymidis are regulated by circulating testosterone, whereas testicular factors secreted into the lumen regulate genes expressed in the IS (lumicrine regulation) [38,39]. Our results show that gonadectomy has an effect on *Pate *gene expressions, although long term effects were detected only in selected members. The qRT-PCR results are presented in Figure 5. *Pate-H, Pate-P, Pate-Q *and *Sslp1*, which are not strongly expressed in the proximal epididymis of the intact mice, did not show gain of expression after gonadectomy (data not shown).

Interestingly, 8 h after gonadectomy the expression of most of the *Pate *genes were up-regulated 2- to 4-fold, with the exception of *Pate*, which was down-regulated. However, in most cases the expression returned to pre-gonadectomy levels 1 day after gonadectomy. The initial increase in expression is associated with the sudden loss of testosterone and suggests partial regulation by androgens, but the subsequent normalization of expression levels indicates that androgens are not sole regulators of the genes. Although unlikely, the possibility that the surgery itself affects gene expression can not be excluded. Only *Pate-C *and *Pate-N *mRNA levels were significantly up- and down-regulated, respectively, 1 day after gonadectomy (P = 0.009 and P = 0.033). Explicit lumicrine regulation was detected for *Pate-A *and *Pate-N*, whose expression was reduced to close to the detection limit 7 days after gonadectomy in mice receiving exogenous testosterone (P < 0.001 and P = 0.005, respectively). A 2-fold reduction was similarly detected for *Pate-X *and *Acrv1 *(P = 0.012 and P = 0.045, respectively). *Pate-A *and *Pate-N *are expressed in the IS at higher levels than in the caput, and, thus, these data well agree with current models of lumicrine regulation of epididymal genes predominantly expressed in the IS [38,39].

## Conclusions

Members of the *Pate *family are predominantly expressed in reproductive organs in male mice, and with the exceptions of *Pate-P *and *Pate-Q *all members studied showed strong expression in the epididymis. The family consists of at least 14 expressed members, including *Acrv1, Sslp1 *and the previously uncharacterized gene (*9230113P08Rik*) which we named *Pate-X*, putatively coding for a PATE family protein. Our studies show that gonadectomy affects the expression of most *Pate *genes, and that *Pate-A *and *Pate-N *are regulated by lumicrine testicular factors. The *Pate *family genes code for putatively secreted, cysteine-rich proteins with a TFP/Ly-6/uPAR domain. Similar proteins are present in venoms of several reptiles, and they are thought to mediate their effects by regulating certain ion channels. The structure and predominant epididymal expression suggest that PATE proteins may function as anti-microbial peptides in the epididymal luminal fluid. The family has also been characterized in the human (Table 3 and [15,17]), and its members may have clinical relevance in epididymal infections and in sperm maturation and fertility.

## Competing interests

The authors declare that they have no competing interests.

## Authors' contributions

HTT planned and performed the *in silico *analyses and gene expression studies, and drafted the manuscript. DAP and AED planned and performed the Northern hybridization experiments and participated in characterization of gene structure and function. IB participated in gene regulation studies and interpretation of the results. PS, IH and MP contributed to the intellectual and experimental design of the study, supervised the experimental work and participated in revising and writing the manuscript. All authors have read and approved the final manuscript.
